# Depletion of the miR-34a “sponge” MALAT1 in aging skeletal muscle: Implications for age-related muscle loss

**DOI:** 10.1093/geroni/igab046.2568

**Published:** 2021-12-17

**Authors:** Ling Ruan, Mark Hamrick, Bharati Mendhe, Carlos Isales, William Hill, Meghan McGee-Lawrence, Sadanand Fulzele

**Affiliations:** 1 Medical College of Georgia, Augusta, Georgia, United States; 2 Medical University of South Carolina, Charleston, South Carolina, United States; 3 Medical College of Georgia, Augusta University, Augusta, Georgia, United States

## Abstract

We have recently shown that increased levels of reactive oxygen species (ROS) in aging skeletal muscle are associated with increased expression of the senescence-associated microRNA miR-34a-5p (miR-34a). The histone deacetylase Sirt1 is a validated target of miR-34a, and miR-34a expression is induced by the tumor suppressor p53 which is itself stimulated by ROS. Long noncoding RNAs (lncRNAs) are known to function as “sponges” for microRNAs, but the role of such competing endogenous RNAs (ceRNA) in muscle aging is not well understood. We therefore examined in skeletal muscles of young (4-6 mos) and aged (22-24) male and female mice the expression of several lncRNAs that are predicted to bind miR-34a-5p in silico and whose predicted binding has been validated experimentally. Results indicate a significant decrease in lncRNA MALAT1 expression with aging. MALAT1 is known to be highly expressed during the later stages of myoblast differentiation and myotube maturation. We therefore treated C2C12 cells at 48 hrs with hydrogen peroxide (10 uM) and examined changes in MALAT1 expression. MALAT1 was significantly decreased with H2O2 treatment, whereas miR-34a is increased in C2C12 cells after hydrogen peroxide exposure. Age-related muscle atrophy mediated by ROS may therefore result in part from related mechanisms involving miR-34a activity: an increase in miR-34a targeting Sirt1 resulting from p53 activation and an increase in miR-34a bioavailability resulting from a decline in miR-34a “sponging” due to ceRNA MALAT1 depletion. These findings suggest that therapeutic interventions increasing MALAT1 expression in muscle may potentially enhance the preservation of muscle mass with aging.

